# Safety assessment of gemtuzumab ozogamicin: real-world adverse event analysis based on the FDA Adverse Event Reporting System

**DOI:** 10.3389/fmed.2025.1643780

**Published:** 2025-09-11

**Authors:** Xuexue Liu, Fang Wu, Zhuoyan Li, Chenxi Li, Xinyu Li, Yining Yuan, Xinyu Sun, Ying Du, Xiao Du, Siliang Wang, Peipei Xu

**Affiliations:** ^1^Department of Hematology, Nanjing Drum Tower Hospital Clinical College of Nanjing University of Chinese Medicine, Nanjing, China; ^2^Department of Pharmacy, Nanjing Drum Tower Hospital, School of Basic Medicine and Clinical Pharmacy, China Pharmaceutical University, Nanjing, China; ^3^Department of Pharmacy, Nanjing Drum Tower Hospital Clinical College of Nanjing University of Chinese Medicine, Nanjing, China; ^4^Department of Hematology, Nanjing Drum Tower Hospital, Affiliated Hospital of Medical School, Nanjing University, Nanjing, China; ^5^Department of Hematology, Nanjing Drum Tower Hospital Clinical College of Nanjing Medical University, Nanjing, China; ^6^Department of Pharmacy, Nanjing Medical Center for Clinical Pharmacy, Nanjing Drum Tower Hospital, Affiliated Hospital of Medical School, Nanjing University, Nanjing, China

**Keywords:** gemtuzumab ozogamicin, adverse events, FAERS, signal detection, acute myeloid leukemia

## Abstract

**Objective:**

To mine adverse drug events (ADEs) following the use of gemtuzumab ozogamicin based on the FDA Adverse Event Reporting System (FAERS), and to provide references for the safety assessment of clinical drug use.

**Methods:**

We obtained reports of adverse events with gemtuzumab ozogamicin as the main suspect from FAERS from within the first quarter of 2004 and the third quarter of 2024. The reporting odds ratio (ROR), comprehensive standard method (MHRA), Bayesian confidence propagation neural network (BCPNN) and multi-item gamma Poisson shrinker (MGPS) were applied to identify AE signals.

**Results:**

A total of 2,065 patients were extracted. Screening for adverse drug events identified 238 positive signals. Among these, febrile neutropenia had the highest number of reports, while liver veno-occlusive disease (VOD) had the strongest signal intensity. Notably, our analysis revealed new high-risk signals, including cryptogenic organizing pneumonia (COP) and increased fibrin degradation products, which were not previously highlighted in FAERS analyses. These findings underscore the need for clinicians to closely monitor patients for these emerging risks, particularly in high-risk populations.

**Conclusion:**

By leveraging extensive real-world evidence from FAERS, we detected previously unidentified AEs linked to gemtuzumab ozogamicin via disproportionality analysis. Our results highlight the necessity for clinicians and pharmacists to prioritize the effective handling of this agent’s high-risk AEs, enhance its rational use in clinical settings, and safeguard patient pharmacotherapy. The identification of new signals, such as COP and increased fibrin degradation products, underscores the importance of continuous pharmacovigilance and the need to update clinical guidelines to reflect these emerging risks.

## 1 Introduction

Acute myeloid leukemia (AML) is a malignant clonal disease characterized by the proliferation of myeloid blasts with impaired differentiation, often leading to impaired normal hematopoiesis and resulting in life-threatening cytopenias and transfusion dependency (1). Traditional treatment strategies have primarily included hematopoietic stem cell transplantation, high-dose cytarabine, and supportive care (2, 3). In 2017, the U.S. Food and Drug Administration (FDA) approved 12 new drugs for various indications of acute myeloid leukemia (AML), which became a turning point in AML research (2). Among them, gemtuzumab ozogamicin (GO), the first approved antibody-drug conjugate (ADC), has revolutionized the treatment landscape of CD33-positive acute myeloid leukemia (4).

Gemtuzumab ozogamicin is composed of a humanized anti-CD33 monoclonal antibody covalently linked to the cytotoxic agent N-acetyl-γ-calicheamicin via a chemical linker (5). CD33, a transmembrane glycoprotein, is found on the surface of myelomonocytic precursor cells and bone marrow myeloblasts in over 80% of AML patients. Patients with high CD33 expression have poorer overall survival (OS), and Cox survival regression analysis indicates that CD33 is an independent prognostic marker (6). Specific CD33 targeting does not impact the maturation of normal CD33–CD34^+^ myeloid progenitor cells, allowing for the recovery of normal non-clonal hematopoiesis (7). After binding to CD33, the drug is rapidly internalized, and calicheamicin is released in the acidic environment of the lysosome. It then binds to DNA, causing double-strand breaks and subsequent cell death (7).

Although gemtuzumab ozogamicin has demonstrated significant therapeutic efficacy in treatment, it still inevitably encounters the challenges commonly faced by antibody-drug conjugates (ADCs) in clinical practice, including drug toxicity, complex pharmacokinetic properties, immunogenicity issues, and drug resistance. Additionally, due to safety concerns, gemtuzumab ozogamicin was withdrawn from the market in 2010. It was re-launched in 2017 based on new clinical trial data, adjusted indications, and unmet clinical needs. Therefore, continuous monitoring of adverse reactions, guiding clinical practice, and updating the product insert are of great significance.

In recent years, drug safety analyses based on FAERS data have been continuously deepening. However, previous studies have mostly focused on known high-risk adverse events such as liver VOD and coagulation abnormalities. Nevertheless, this study, through in-depth mining of FAERS data, has for the first time identified signals related to gemtuzumab ozogamicin, such as cryptogenic organizing pneumonia (COP) and increased fibrin degradation products, which are new high-risk signals. These newly discovered signals indicate that in clinical practice, we need to pay more attention to patients’ coagulation function and pulmonary health, especially in high-risk patients, such as elderly patients, those with a history of coagulation dysfunction, or a history of liver disease. These additional high-risk signals not only provide clinicians with a more comprehensive understanding of drug safety but also offer important basis for optimizing the clinical use strategy of gemtuzumab ozogamicin and reducing the risk of adverse events. This study aims to extract and analyze relevant data from the FAERS database to mine, organize, and analyze adverse drug event (ADE) signals related to gemtuzumab ozogamicin, in order to reduce its usage risks and provide references for rational clinical drug use.

## 2 Materials and methods

### 2.1 Data sources and processing

The data used for this study was sourced from the FDA Adverse Event Reporting System (FAERS), which is publicly accessible on the FDA’s official website. The FAERS database has been available since the first quarter of 2004 and is updated quarterly. It offers two formats for download: ASCII data packages and XML data packages. For this analysis, the original ASCII data package was downloaded and processed using R version 4.4.2.

To address data quality in FAERS, which is based on voluntary reporting and thus prone to duplicates or withdrawn reports, we meticulously followed the FDA’s data-cleaning protocols. Deduplication was conducted using PRIMARYID, CASEID, and FDA_DT from the DEMO table. The reports were sorted by CASEID, FDA_DT, and PRIMARYID. For entries sharing the same CASEID, the most recent FDA_DT report was retained. In cases where both CASEID and FDA_DT matched, the entry with the highest PRIMARYID was kept. Furthermore, starting in the first quarter of 2019, each quarterly data package has included a deletion list. Post deduplication, reports flagged for deletion by their CASEID were removed from the dataset, ensuring the analysis data was accurate and representative (8).

### 2.2 Handling false-positive adverse events

The FAERS database contains a wide range of drugs and their related information, aiming to investigate the associations between drugs and diseases to provide guidance for rational clinical drug use. However, in actual clinical practice, patients often take multiple medications. When patients are on polypharmacy, studying the relationship between a specific drug and a specific adverse event (AE) significantly increases the complexity of the research. For instance, in a report that includes five medications and four adverse reactions, assuming that one adverse reaction is caused by one drug alone would lead to a large number of false-positive “drug-adverse event” reports.

**Figure 1:**
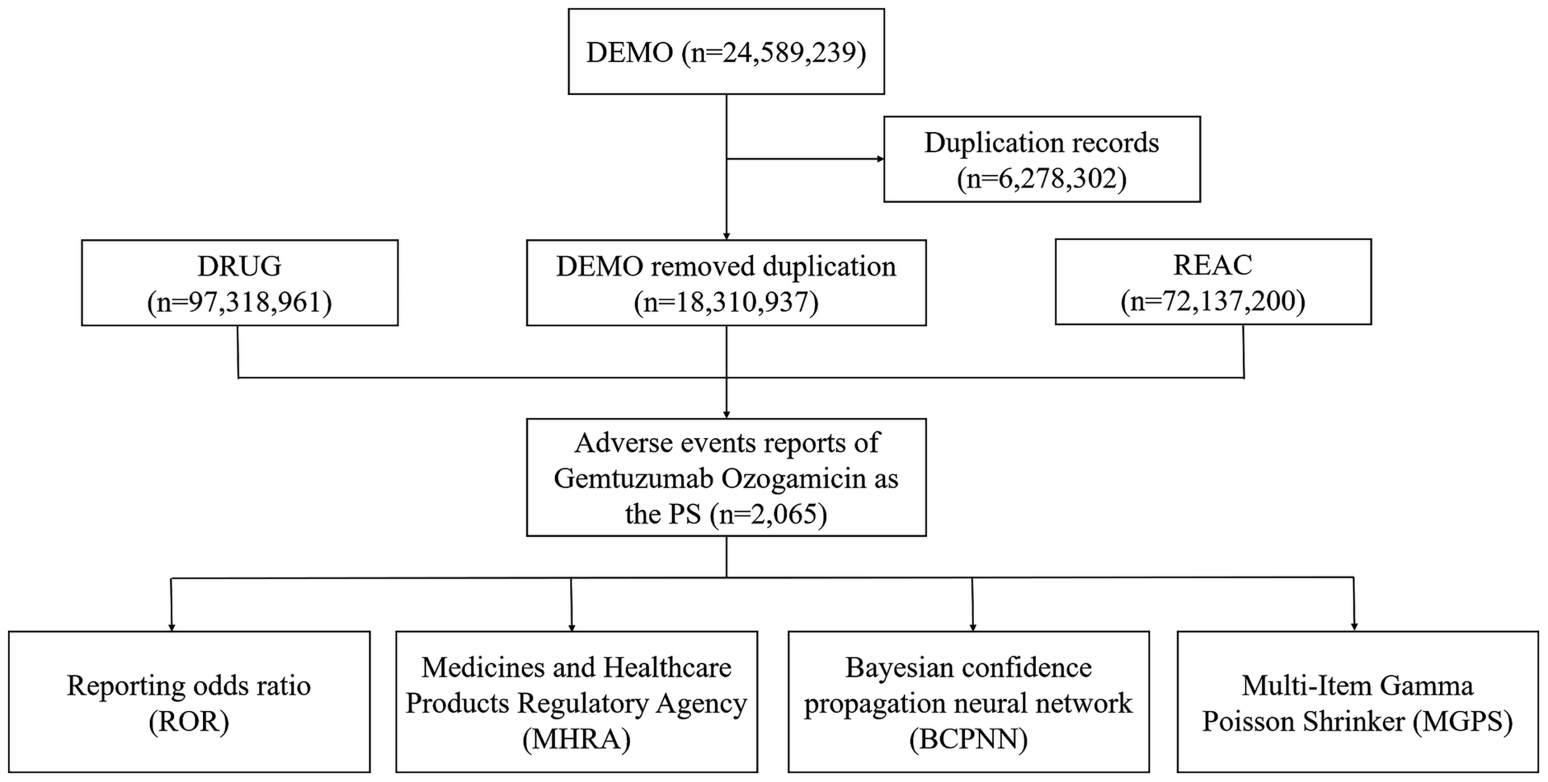
Flow diagram of the study (2004Q1–2024Q3) (DEMO, demo graphic and administrative information; DRUG, drug information; REAC, preferred terminology for adverse drug reactions; PS, primary suspect drug).

To address this issue, the database has added a new field called role_cod in the DRUG table. This field helps identify true “drug-adverse event” signals by categorizing the role of the drug in the reported event into four types: primary suspect (PS), secondary suspect (SS), concomitant (C), and interaction (I). In this study, we identified cases in the DRUG file by searching both the generic name (gemtuzumab ozogamicin) and the brand name (Mylotarg). To enhance accuracy, we focused on cases where the role_cod was marked as PS. Additionally, to reduce the false-positive rate, we applied disproportionality analysis to mine true “drug-adverse event” signals. During the study period, 24,589,239 reports related to gemtuzumab ozogamicin were obtained from the FAERS database. After excluding duplicates, 18,310,937 reports identified gemtuzumab ozogamicin as the PS, and 2,065 ADRSs were associated with gemtuzumab ozogamicin (Figure 1).

### 2.3 Statistical analysis

In pharmacovigilance research, disproportionality analysis has garnered widespread attention. This method compares the proportion of specific adverse reactions associated with a single drug or multiple drugs to the proportion of adverse reactions reported for the same drug in the entire database. Building on disproportionality analysis, we further employed disproportionality measures to identify associations between drugs and AEs. The reporting odds ratio (ROR), comprehensive standard method (MHRA), Bayesian confidence propagation neural network (BCPNN), and multi-item gamma Poisson shrinker (MGPS) algorithms are four major specific indicators used to evaluate signals of AEs related to the target drug. These four methods are based on a 2 × 2 contingency table and involve a statistical analysis approach (Table 1). To evaluate the statistical relationship between a specific drug and a specific adverse event (AE), researchers calculate the relative frequency of the target adverse reaction caused by the target drug in the database over a defined period. This method helps determine whether there is a significant association between the drug and the AE. Generally speaking, if the frequency of AEs related to the target drug in the database is high and reaches a certain critical value, it indicates that the drug has generated a signal for the AE (Table 2). The signal generation conditions for the ROR algorithm are: the lower limit of the 95% confidence interval (CI) is greater than 1, and a ≥ 3. The signal generation conditions for the MHRA algorithm are: a ≥ 3, PRR (proportional reporting ratio) is greater than 2, and *χ*^2^ ≥ 4. The signal generation condition for the BCPNN (Bayesian confidence propagation neural network) algorithm is: IC (information component) minus 2 standard deviations (SD) is greater than 0. The standard for judging drug adverse reaction signals by the MGPS (multi-item gamma Poisson shrinker) algorithm is EBGM05 > 2, where EBGM05 represents the lower limit of the 95% confidence interval of the empirical Bayes geometric mean (EBGM).

**Table 1:** Fourfold table of disproportionality method.

Drug category	Event of interest	All other events
Target drug	a	b
Gemtuzumab ozogamicin	c	d

a represents the number of reports that include both the target drug and its associated adverse reaction. b indicates the number of reports that mention the target drug but involve other adverse reactions. c refers to the number of reports that mention the associated adverse reaction but involve other drugs. d denotes the number of reports that include neither the target drug nor the associated adverse reaction.

**Table 2:** Calculation formula and standard of signal detection.

Algorithm	Calculation formula	Criterion
ROR	$\mathrm{ROR}=\frac{\mathrm{ad}}{\mathrm{bc}}$ $\mathrm{SE}(\ln\mathrm{ROR})=\sqrt{\left(\frac{1}{a}+\frac{1}{b}+\frac{1}{c}+\frac{1}{d}\right)}$ $95\%\mathrm{CI}=e^{\ln(\mathrm{ROR})\pm \sqrt[{1.96}]{\left(\frac{1}{a}+\frac{1}{b}+\frac{1}{c}+\frac{1}{d}\right)}}$	a ≥ 3 95% CI (lower limit) > 1
MHRA	$\mathrm{PRR}=\frac{a/(a+b)}{c/(c+d)}$ χ2=[(ad−bc)^2](a+b+c+d)/[(a+b)(c+d)(a+c)(b+d)]	a ≥ 3, PRR > 2 and *χ*^2^ ≥ 4
BCPNN	$\begin{array}{l} \mathrm{IC}=\log_{2}\frac{a(a+b+c+d)}{\left(a+c)(a+b\right)}\\ E(\mathrm{IC})=\log_{2}\frac{\left(a+\gamma 11)(a+b+c+d+\alpha )(a+b+c+d+\beta \right)}{\left(a+b+c+d+\gamma )(a+b+\alpha 1)(a+c+\beta 1\right)}\\ V(\mathrm{IC})=\frac{1}{{\left(\ln2\right)}^{2}}\{[\frac{(a+b+c+d)-a+\gamma +\gamma 11}{\left(a+\gamma 11)(1+a+b+c+d+\gamma \right)}]\\ +[\frac{(a+b+c+d)-(a+b)+\alpha -\alpha 1}{\left(a+b+\alpha 1)(1+a+b+c+d+\alpha \right)}]\\ +[\frac{(a+b+c+d)-(a+c)+\beta -\beta 1}{\left(a+c+\beta 1)(1+a+b+c+d+\beta \right)}]\} \end{array}$ γ=γ11(a+b+c+d+α)(a+b+c+d+β)(a+b+α1)(a+c+β1)IC−2SD=E(IC)−2V(IC)^0.5	IC-2SD > 0
MGPS	$\begin{array}{l} \text{EBGM}=a(a+b+c+d)/(a+c)/(a+b)\\ 95\%\mathrm{CI}=e^{\ln(\text{EBGM})\pm \sqrt[{1.96}]{\left(\frac{1}{a}+\frac{1}{b}+\frac{1}{c}+\frac{1}{d}\right)}} \end{array}$	EBGM05 > 2

## 3 Results

### 3.1 General characteristics

A total of 2,065 patients with gemtuzumab ozogamicin as the primary suspect drug were retrieved, and the results of data extraction are shown in Figure 1. Among the cases with known gender, there were 953 male patients (46.15%) and 765 female patients (37.04%). The most common age group was 45–64 years (26.59%). The majority of reports originated from the United States (32.69%), and most reporters were physicians (50.51%). Hospitalization was the primary outcome (30.91%), as shown in Table 3.

**Table 3:** Characteristics of reports associated with gemtuzumab ozogamicin.

Characteristics	Case number	Proportion (%)
Gender		
Male	953	46.15
Female	765	37.05
Unknown	347	16.8
Age (years)		
<18	80	3.87
18–44	236	11.43
45–64	549	26.59
65–75	360	17.43
>75	163	7.89
Unknown	677	32.78
Reporter		
Medical doctor	1,043	50.51
Other	212	10.27
Pharmacist	210	10.17
Consumer	40	1.94
Reported countries		
United States	675	32.69
United Kingdom	257	12.45
France	235	11.38
Japan	232	11.23
Italy	96	4.65
Outcomes		
Hospitalization	1,743	30.91
Other	1,472	26.1
Death	1,349	23.92
Life-threatening	939	16.65
Disability	51	0.9
Required intervention to prevent permanent impairment/damage	5	0.09

### 3.2 ADE risk signals

Gemtuzumab ozogamicin was identified as the primary suspect drug, with a total of 9,455 drug adverse event (ADE) reports extracted, involving 2,065 patients. After screening using the ROR, MHRA, BCPNN, and MGPS method, a total of 238 valid PT signals were identified, with 6,198 related ADE reports. The 238 valid signals were ranked according to the lower limit of the 95% CI of ROR. The top 30 PTs in signal intensity are shown in Table 4. The top 10 are liver VOD, increased fibrin degradation products, refractoriness to platelet transfusion, pneumonia due to aspergillus, increased blast cell count, VOD, bacteria identified in blood, central line infection, serratia sepsis, and perianal abscess.

**Table 4:** The signal intensity ranking of the ADE signals for gemtuzumab ozogamicin.

Sorting	PT	*N*	ROR (95% CI)	PRR (*χ*^2^)	IC025/IC-2SD	EBGM05
1	Liver VOD	161	230.83 (196.94, 270.55)	226.91 (34842.15)	6.31	186.29
2	Fibrin degradation products increased	15	322.91 (191.9, 543.38)	322.4 (4551.35)	3.19	181.47
3	Refractoriness to platelet transfusion	4	237.64 (87.4, 646.09)	237.54 (904.85)	0.97	83.92
4	pneumonia aspergillus	3	265.94 (83.57, 846.3)	265.86 (756.66)	0.5	79.87
5	Blast cell count increased	17	110.72 (68.49, 178.99)	110.52 (1810.35)	3.27	67.09
6	VOD	36	92.88 (66.78, 129.19)	92.53 (3208.21)	4.25	65.49
7	Bacteria blood identified	4	175.96 (65.05, 475.95)	175.88 (674.96)	0.97	63.11
8	Central line infection	8	127.05 (63.04, 256.07)	126.95 (978.09)	2.11	61.64
9	Serratia sepsis	3	176.39 (55.91, 556.5)	176.33 (507.48)	0.5	54.24
10	Perianal abscess	3	163.08 (51.76, 513.84)	163.03 (469.8)	0.5	50.32
11	Caecitis	7	88.88 (42.12, 187.55)	88.81 (598.55)	1.86	41.46
12	Neutrophil percentage decreased	7	86.96 (41.22, 183.49)	86.9 (585.55)	1.86	40.58
13	Blast cells present	6	79.68 (35.59, 178.4)	79.63 (459.53)	1.6	35.09
14	Neutropenic colitis	16	54.66 (33.39, 89.46)	54.57 (833.5)	3.01	33.03
15	Acute myeloid leukaemia	93	39.94 (32.54, 49.03)	39.56 (3472.32)	4.5	32.01
16	Febrile neutropenia	342	36.02 (32.32, 40.13)	34.75 (11154.52)	4.82	31.0
17	Intravascular haemolysis	6	68.48 (30.61, 153.2)	68.44 (394.04)	1.59	30.24
18	Blood culture positive	23	46.1 (30.57, 69.51)	45.99 (1004.21)	3.4	30.26
19	Aspergillosis	12	52.35 (29.64, 92.45)	52.29 (598.25)	2.6	29.35
20	Bacteria stool identified	3	88.65 (28.34, 277.3)	88.62 (255.95)	0.49	27.9
21	Cryptogenic organizing pneumonia	3	88.65 (28.34, 277.3)	88.62 (255.95)	0.49	27.9
22	Catheter-related infection	9	50.58 (26.23, 97.51)	50.53 (433.14)	2.17	25.98
23	Blood fibrinogen increased	7	53.8 (25.55, 113.28)	53.76 (359.12)	1.8	25.3
24	Neutropenic infection	8	51.19 (25.51, 102.7)	51.15 (389.86)	1.99	25.27
25	Pneumonia fungal	21	38.68 (25.17, 59.44)	38.6 (764.02)	3.21	24.95
26	Neutropenic sepsis	36	32.07 (23.09, 44.52)	31.95 (1073.4)	3.64	22.89
27	Enterococcal bacteremia	7	46.87 (22.27, 98.63)	46.83 (311.44)	1.77	22.08
28	Streptococcal bacteremia	7	45.8 (21.76, 96.38)	45.77 (304.12)	1.77	21.58
29	Blood bilirubin increased	108	25.62 (21.19, 30.99)	25.34 (2515.1)	4.09	20.87
30	Liver tenderness	3	65.73 (21.06, 205.15)	65.71 (189.01)	0.48	20.82

### 3.3 Distribution and proportion of ADE signals across SOC

**Figure 2:**
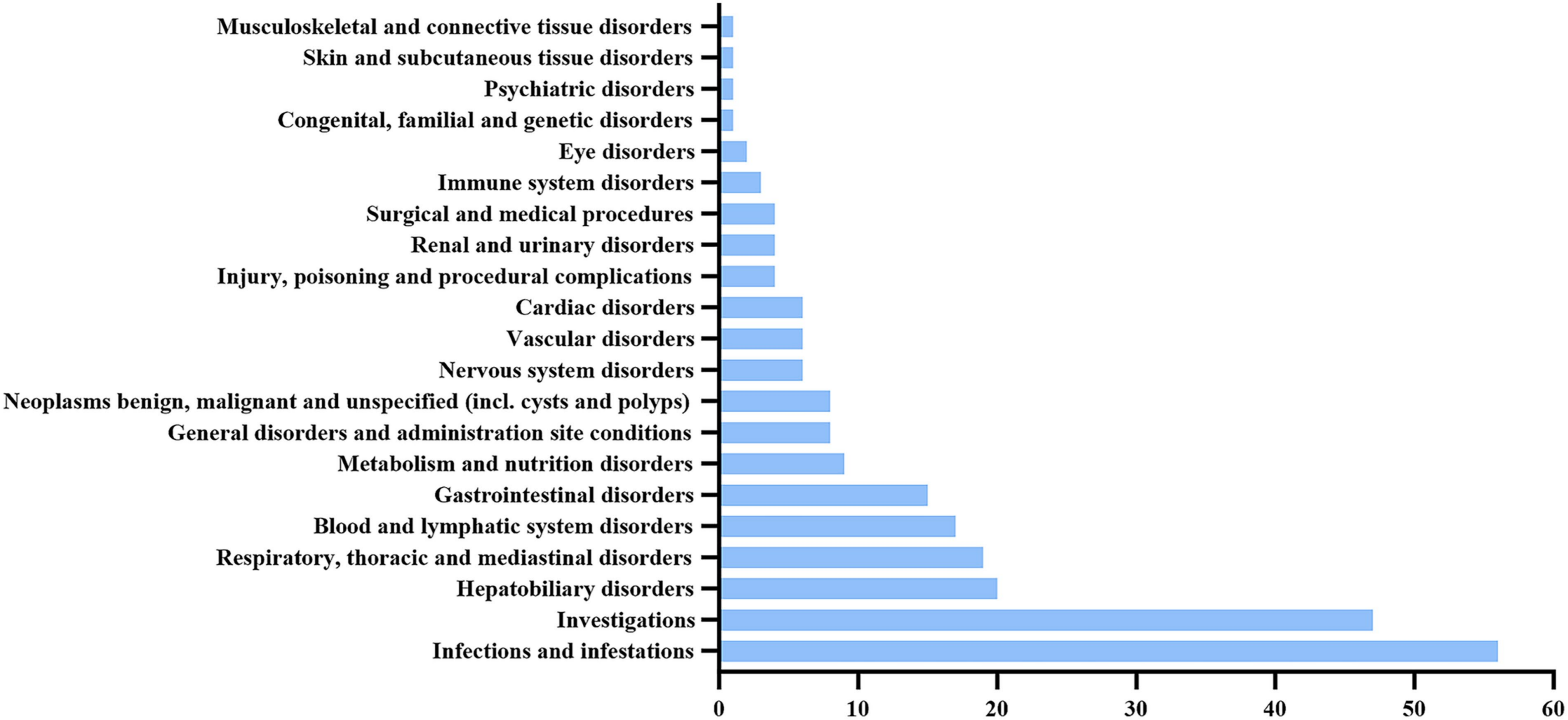
The SOC involved in the ADE reports of gemtuzumab ozogamicin.

The 238 valid positive PT (preferred term) signals obtained were categorized, involving a total of 21 SOC (Figure 2). The PTs were ranked according to the number of reports, with the top 30 listed in Table 5. The top five SOC categories are blood and lymphatic system disorders, general disorders and administration site conditions, infections and infestations, investigations, and hepatobiliary disorders.

**Table 5:** Signal results of gemtuzumab ozogamicin ADE reports by major systems involved in the top 30 (*n* = 6,198).

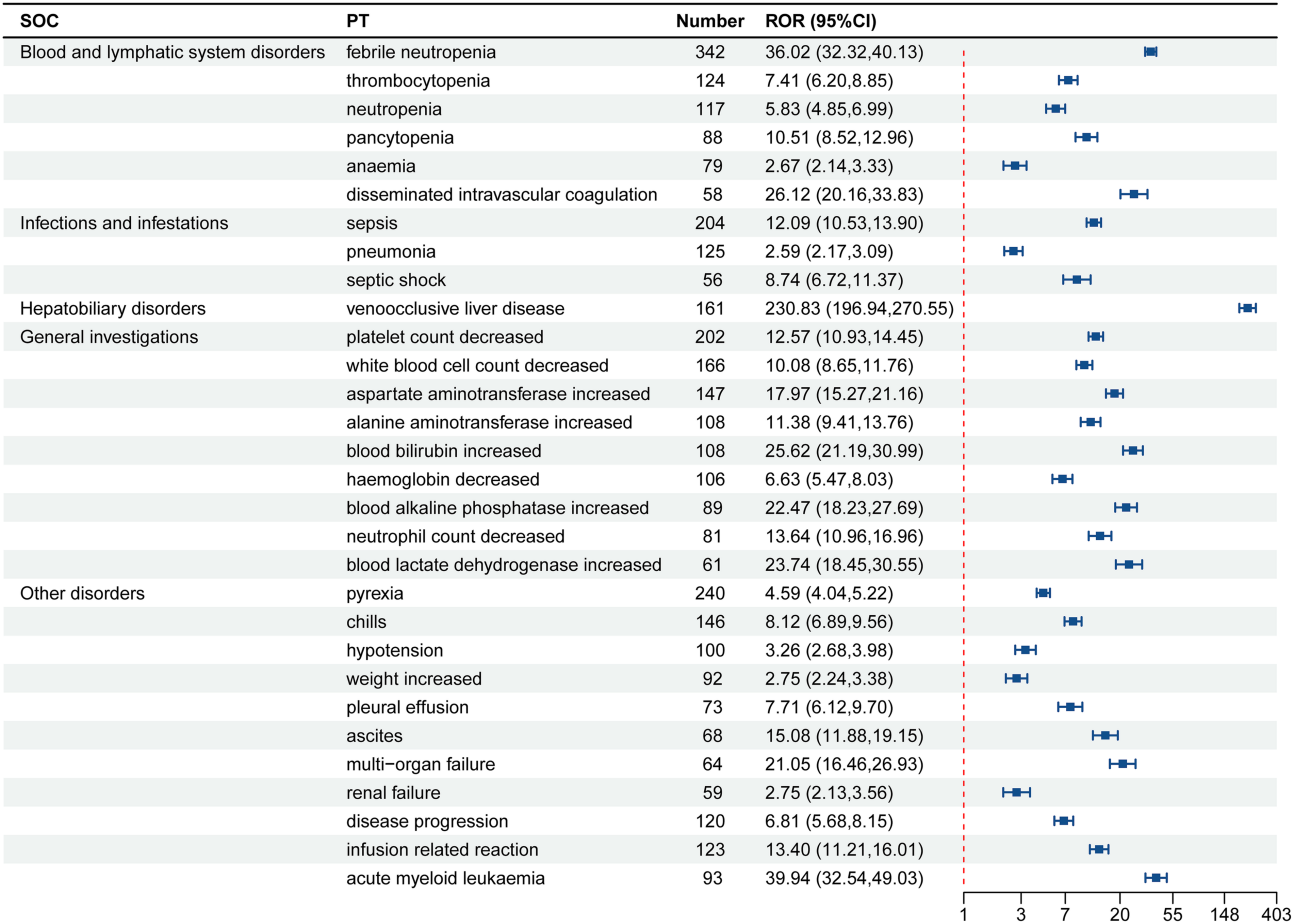

## 4 Discussion

In this study, among the 2,065 patients associated with gemtuzumab ozogamicin, the gender distribution showed that males accounted for a slightly higher proportion than females (46.15% vs. 37.05%), which is consistent with the epidemiological characteristics of acute myeloid leukemia (AML) (9). In terms of age distribution, patients aged 45–64 years had the highest proportion (26.59%), followed by those aged 65–75 years (17.43%). This trend aligns with the significant increase in AML incidence with age (9). However, the proportion of younger patients aged 18–44 years was 11.43%, suggesting that gemtuzumab ozogamicin may be used in younger patients with relapsed/refractory AML, high CD33 expression, or specific molecular subtypes. Regarding the reporting countries, the United States accounted for 32.69% of the total reports, significantly higher than other countries (e.g., the United Kingdom at 12.44%). The lower reporting rates in countries such as Japan and France may be limited by drug accessibility or differences in regulatory reporting culture, indicating potential geographical bias in the study results. Among the reporters, physicians accounted for 50.51%, highlighting their key role in drug safety monitoring but also revealing the insufficient involvement of other professions, such as pharmacists. In terms of patient outcomes, hospitalization (30.91%) was the most common outcome, suggesting that clinicians need to carefully balance efficacy and safety. Additionally, death (23.92%) and life-threatening events (16.65%) had relatively high proportions, further emphasizing the necessity of clinical risk management for this drug. Future efforts should focus on optimizing treatment protocols through precise dose adjustments, toxicity prevention strategies, and integration of real-world data to reduce hospitalization rates and improve patient quality of life.

Hepatotoxicity has always been a significant factor limiting the development of gemtuzumab ozogamicin, especially the occurrence of severe and potentially fatal liver VOD (10). This condition is primarily characterized by fibrotic occlusion of the small hepatic veins (especially the sinusoids), leading to impaired hepatic blood flow and liver function damage. It is listed as a black box warning in the product insert. In this study, this signal was most strongly associated with gemtuzumab ozogamicin, with a total of 161 liver VOD cases reported. The reporting odds ratio (ROR) was 230.83 (95% confidence interval, 196.94–270.55), indicating the highest signal strength, which is consistent with the product insert. In this study, we found that the signal strength of “increased fibrin degradation products” (ROR = 322.91) was higher than that of liver VOD (ROR = 230.83). This may suggest that we need to pay more attention to the clinical significance of increased fibrin degradation products. Increased fibrin degradation products may be an early sign of liver VOD and may also reflect other types of liver injury or coagulation disorders. Changes in fibrinolytic and coagulation parameters can facilitate the early diagnosis of liver VOD. For example, a study by Sartori et al. (11) demonstrated that children with liver VOD had a significant increase in plasminogen activator inhibitor-1 (PAI-1), tissue plasminogen activator (t-PA), and D-dimer levels two days before the clinical diagnosis of liver VOD. These changes indicate that fibrin degradation products could serve as early indicators of liver VOD development. Clinicians should closely monitor patients’ coagulation function indicators, especially for those with a history of liver disease or coagulation disorders, to detect and manage potential hepatotoxicity risks in a timely manner.

In addition, the risk of hepatotoxicity may be further exacerbated in elderly patients. Elderly patients typically have lower hepatic functional reserve and reduced capacity for drug metabolism and clearance, which may lead to the accumulation of gemtuzumab ozogamicin and its metabolites in the body, increasing the probability of developing liver VOD. Moreover, elderly patients often use other hepatotoxic drugs concurrently (such as certain antibiotics, lipid-lowering drugs, etc.), which may produce synergistic hepatotoxic effects with gemtuzumab ozogamicin, further aggravating liver injury. Therefore, in the treatment of elderly patients, special attention should be paid to liver function monitoring. It is recommended to conduct a comprehensive liver function assessment for elderly patients before treatment, including biochemical liver function indicators, coagulation function, and liver imaging examinations. During the treatment process, liver function indicators (such as ALT, AST, bilirubin, prothrombin time, etc.) should be regularly monitored, and the treatment plan should be adjusted in a timely manner based on the monitoring results. For patients with abnormal liver function, hepatoprotective treatment should be given in a timely manner, and the use of gemtuzumab ozogamicin should be considered to be suspended or discontinued. Moreover, the concurrent use of other hepatotoxic drugs should be avoided as much as possible to reduce the risk of liver injury (12).

Another risk signal uncovered in this study, tenderness in the liver area, has a clear clinical correlation with liver VOD. The core pathological change of liver VOD is the injury of endothelial cells in the small hepatic veins or hepatic sinusoids, leading to vascular obstruction and impaired blood flow within the liver, which in turn causes hepatic congestion, hepatocyte necrosis, and portal hypertension. During this process, the liver may become enlarged due to congestion and inflammatory response, manifesting as tenderness in the liver area upon palpation (13). Therefore, tenderness in the liver area may be one of the direct clinical manifestations of liver VOD and can serve as one of the early warning signs of liver VOD induced by gemtuzumab ozogamicin. Clinical studies by Yu and Liu (7) have shown that fractionated low-dose treatment significantly reduces the risk of hepatotoxicity associated with gemtuzumab ozogamicin.

There were 342 reports of febrile neutropenia (ROR = 36.02), with a significantly high signal strength, making it the most common infection-related event. The product insert has already clearly listed it as a common adverse reaction, which is directly related to drug-induced bone marrow suppression (neutropenia). Clinical research by sylvie castaigne also showed that the incidence of neutropenia and grade 3 or 4 infections in the gemtuzumab ozogamicin group was slightly higher than in the treatment group receiving daunorubicin combined with cytarabine, indicating that gemtuzumab ozogamicin may increase the risk of infection (14).

There were 3 reports of pneumonia aspergillus (ROR = 265.94), with an extremely high signal strength. The product insert only mentions the risk of “opportunistic infections” without specifically listing aspergillus infections. It is recommended to supplement the product insert with specific pathogen risk warnings. In response to the high-risk infection event of pulmonary aspergillosis, the following specific preventive measures are recommended for high-risk patients (such as those with prolonged neutropenia or combined immunodeficiency): (1) Antifungal drug selection: voriconazole is recommended as the first-line prophylactic drug. Adverse drug reactions, especially liver function abnormalities, should be closely monitored during treatment. (2) Monitoring frequency: Weekly chest imaging examinations (such as chest CT) and fungal biomarker tests (such as galactomannan antigen detection) should be performed to detect early signs of infection. (3) For patients presenting with fever or respiratory symptoms, sputum and blood cultures should be immediately performed, and empirical broad-spectrum antifungal drugs should be used before the results are available. These specific measures can effectively reduce the incidence and mortality of pulmonary aspergillosis. There were 8 reports of central line infection (ROR = 127.05), indicating a risk of catheter-related infections. Strict aseptic techniques should be applied during clinical procedures, catheters should be regularly replaced, and local infection signs should be monitored. For infections such as serratia sepsis, Enterococcus bacteremia, Streptococcus bacteremia, perianal abscess, cecitis, neutropenic colitis, and cryptogenic organizing pneumonia, gemtuzumab ozogamicin may increase the risk of opportunistic infections by destroying CD33-positive immune cells (such as monocytes) and further weakening the innate immune response (15). Clinical management should focus on infection prevention, such as using fluoroquinolones (e.g., levofloxacin) to prevent bacterial infections during neutropenia (ANC < 500/mm^3^) (16). For high-risk patients (such as those with prolonged neutropenia), antifungal drugs (e.g., voriconazole) should be added, and early identification and intervention should be implemented.

As a treatment for acute myeloid leukemia (AML), gemtuzumab ozogamicin has still uncovered the risk signal of AML (ROR = 39.94). The signal of increased AML risk associated with GO may involve the following two scenarios: (1) Relapse of primary AML: Although GO has significant efficacy against CD33-positive AML, some patients may experience relapse after treatment. (2) Therapy-related AML (t-AML): The cytotoxic component of GO, calicheamicin, exerts its anticancer effect by inducing DNA double-strand breaks (17), but it may also damage normal hematopoietic stem cells, leading to increased genomic instability. This genomic instability may promote the accumulation of mutations, thereby increasing the risk of t-AML. Moreover, the depletion of CD33-positive immune cells (such as monocytes) by GO may weaken the patient’s innate immune response, further increasing the risk of infection and indirectly promoting the expansion of leukemia clones. Studies have shown that TP53 and FLT3 mutations are common resistance-related mutations in AML patients (18), and GO treatment may lead to the selection and expansion of these resistant clones, thereby increasing the probability of t-AML occurrence. Therefore, in clinical practice, it is important to closely monitor the hematological indicators and genetic mutation status of patients to detect and manage potential relapse or t-AML risks in a timely manner.

The increase in fibrinogen (ROR = 53.8) has significant statistical strength. Gemtuzumab ozogamicin (GO) may activate the coagulation system by damaging endothelial cells, which in turn leads to the conversion of fibrinogen to fibrin and the formation of blood clots. During this process, the fibrinolytic system is also activated to dissolve the formed blood clots, resulting in an increase in fibrin degradation products (such as D-dimer and fibrin degradation products) (19). This dynamic change in coagulation and fibrinolysis is a protective response of the body to endothelial cell damage, but it may also indicate potential coagulation dysfunction. However, the current product insert does not cover this risk. Clinically, it is necessary to strengthen the monitoring of coagulation function and take preventive measures for high-risk patients. In the future, it is necessary to verify the risk level and improve the warning content of the product insert through mechanism research and real-world data, in order to optimize patient safety management.

This study has certain limitations. The FAERS database relies on voluntary reporting by healthcare providers and patients, which is subject to significant underreporting bias. This bias can lead to an underestimation of the true incidence of adverse events. Studies have shown that only a fraction of adverse events are reported to FAERS, and this fraction can vary widely depending on the type of event and the awareness of healthcare providers. One specific concern is the potential overrepresentation of serious and fatal events in the FAERS database. Healthcare providers may be more likely to report severe or fatal adverse events due to their clinical significance and the potential legal implications. This can lead to an overestimation of the risk of serious adverse events associated with gemtuzumab ozogamicin. For instance, an increase in fibrin degradation products could be caused by concomitant medications or underlying diseases, rather than gemtuzumab ozogamicin itself. It is important to acknowledge that the FAERS database is cross-sectional in nature, which means it cannot track the temporal associations of adverse events. For example, it is not possible to determine the latency period after drug administration or the cumulative dose effects from the data provided by FAERS. This limitation may affect the interpretation of our findings, as it prevents us from establishing a clear temporal relationship between the use of gemtuzumab ozogamicin and the occurrence of adverse events. Future studies should consider incorporating additional data sources or methods that can provide more detailed temporal information to better understand the dynamics of drug safety. The study also did not differentiate patient subgroups (such as elderly, pediatric, or relapsed/refractory AML patients), making it difficult to assess risk profiles in specific populations (20).

## 5 Conclusion

Our study has identified several high-risk adverse events associated with gemtuzumab ozogamicin, including well-known risks such as liver VOD and coagulation abnormalities. Notably, our analysis revealed that increased fibrin degradation products had the highest signal intensity among the identified adverse events. These findings are partially consistent with the current FDA drug label. However, our analysis of FAERS data also uncovered additional risks that are not sufficiently addressed in the current drug label, such as Aspergillus pneumonia and organizing pneumonia.

Given these findings, we recommend that regulatory agencies consider updating the drug label to include these high-risk adverse events. This would enhance the safety and efficacy of gemtuzumab ozogamicin in clinical practice by providing healthcare providers with more comprehensive information to guide patient management.

Clinicians should integrate the recommendations in the drug label with real-world evidence to implement individualized monitoring strategies. This includes enhanced coagulation and pulmonary function assessments, particularly for patients at higher risk. Multidisciplinary collaboration is essential to optimize risk management and ensure patient safety.

Future efforts should focus on promoting dynamic linkage between FAERS data and the drug label. This will enhance the timeliness and precision of pharmacovigilance, allowing for continuous updates to the drug label based on the latest evidence from real-world data. This approach will help healthcare providers stay informed about potential risks and improve patient outcomes.

## Data Availability

Publicly available datasets were analyzed in this study. This data can be found here: https://www.fda.gov/.

## References

[1] DiNardo CD Erba HP Freeman SD Wei AH. Acute myeloid leukaemia. Lancet. (2023) 401:2073–86. doi: 10.1016/S0140-6736(23)00108-3, PMID: 37068505

[2] Kantarjian HM DiNardo CD Kadia TM Daver NG Altman JK Stein EM. Acute myeloid leukemia management and research in 2025. CA Cancer J Clin. (2024) 75:46–67. doi: 10.3322/caac.21873, PMID: 39656142 PMC11745214

[3] Premnath N Madanat YF. Paradigm shift in the management of acute myeloid leukemia-approved options in 2023. Cancers. (2023) 15:3002. doi: 10.3390/cancers15113002, PMID: 37296964 PMC10251983

[4] Oya S Ozawa H Morishige S Maehiro Y Umeda M Takaki Y. High-dose cytarabine plus gemtuzumab ozogamicin as consolidation therapy in patients with favorable- or intermediate-risk acute myeloid leukemia. Int J Hematol. (2024) 120:297–304. doi: 10.1007/s12185-024-03814-z, PMID: 38963637

[5] Jen EY Ko C-W Lee JE Del Valle PL Aydanian A Jewell C. FDA approval: gemtuzumab ozogamicin for the treatment of adults with newly diagnosed CD33-positive acute myeloid leukemia. Clin Cancer Res. (2018) 24:3242–6. doi: 10.1158/1078-0432.Ccr-17-3179, PMID: 29476018

[6] Liu J Tong J Yang H. Targeting CD33 for acute myeloid leukemia therapy. BMC Cancer. (2022) 22:24. doi: 10.1186/s12885-021-09116-5, PMID: 34980040 PMC8722076

[7] Yu B Liu D. Gemtuzumab ozogamicin and novel antibody-drug conjugates in clinical trials for acute myeloid leukemia. Biomark Res. (2019) 7:24. doi: 10.1186/s40364-019-0175-x, PMID: 31695916 PMC6824118

[8] Shi X Wang C Liu X Zou L Guo P. A real-world pharmacovigilance analysis of omadacycline in FDA Adverse Event Reporting System (FAERS) database. Front Pharmacol. (2025) 16:1558868. doi: 10.3389/fphar.2025.1558868, PMID: 40235549 PMC11996809

[9] Zhou Y Huang G Cai X Liu Y Qian B Li D. Global, regional, and national burden of acute myeloid leukemia, 1990–2021: a systematic analysis for the global burden of disease study 2021. Biomark Res. (2024) 12:101. doi: 10.1186/s40364-024-00649-y, PMID: 39256810 PMC11389310

[10] McKoy JM Angelotta C Bennett CL Tallman MS Wadleigh M Evens AM. Gemtuzumab ozogamicin-associated sinusoidal obstructive syndrome (SOS): an overview from the research on adverse drug events and reports (RADAR) project. Leuk Res. (2007) 31:599–604. doi: 10.1016/j.leukres.2006.07.005, PMID: 16959316

[11] Sartori MT Spiezia L Cesaro S Messina C Paris M Pillon M. Role of fibrinolytic and clotting parameters in the diagnosis of liver veno-occlusive disease after hematopoietic stem cell transplantation in a pediatric population. Thromb Haemost. (2005) 93:682–9. doi: 10.1160/th04-09-0621, PMID: 15841312

[12] Woods JD Klepin HD. Geriatric assessment in acute myeloid leukemia. Acta Haematol. (2024) 147:219–28. doi: 10.1159/000535500, PMID: 38035561 PMC10963150

[13] Wadleigh M Ho V Momtaz P Richardson P. Hepatic veno-occlusive disease: pathogenesis, diagnosis and treatment. Curr Opin Hematol. (2003) 10:451–62. doi: 10.1097/00062752-200311000-00010, PMID: 14564177

[14] Castaigne S Pautas C Terré C Raffoux E Bordessoule D Bastie J-N. Effect of gemtuzumab ozogamicin on survival of adult patients with de-novo acute myeloid leukaemia (ALFA-0701): a randomised, open-label, phase 3 study. Lancet. (2012) 379:1508–16. doi: 10.1016/S0140-6736(12)60485-1, PMID: 22482940

[15] Lamba JK Chauhan L Shin M Loken MR Pollard JA Wang YC. CD33 splicing polymorphism determines gemtuzumab ozogamicin response in *de novo* acute myeloid leukemia: report from randomized phase III children’s oncology group trial AAML0531. J Clin Oncol. (2017) 35:2674–82. doi: 10.1200/jco.2016.71.2513, PMID: 28644774 PMC5549451

[16] Alexander S Fisher BT Gaur AH Dvorak CC Villa Luna D Dang H. Effect of levofloxacin prophylaxis on bacteremia in children with acute leukemia or undergoing hematopoietic stem cell transplantation: a randomized clinical trial. JAMA. (2018) 320:995–1004. doi: 10.1001/jama.2018.12512, PMID: 30208456 PMC6143098

[17] Nicolaou KC Pitsinos EN Theodorakis EA Saimoto H Wrasidlo W. Synthetic calicheamicin mimics with novel initiation mechanisms: DNA cleavage, cytotoxicity, and apoptosis. Chem Biol. (1994) 1:57–66. doi: 10.1016/1074-5521(94)90041-8, PMID: 9383371

[18] Jambhekar A Ackerman EE Alpay BA Lahav G Lovitch SB. Comparison of TP53 mutations in myelodysplasia and acute leukemia suggests divergent roles in initiation and progression. Blood Neoplasia. (2024) 1:100004. doi: 10.1016/j.bneo.2024.100004, PMID: 40453522 PMC12082110

[19] Gimbrone MA García-Cardeña G. Endothelial cell dysfunction and the pathobiology of atherosclerosis. Circ Res. (2016) 118:620–36. doi: 10.1161/CIRCRESAHA.115.306301, PMID: 26892962 PMC4762052

[20] Giunchi V Fusaroli M Hauben M Raschi E Poluzzi E. Challenges and opportunities in accessing and analysing FAERS data: a call towards a collaborative approach. Drug Saf. (2023) 46:921–6. doi: 10.1007/s40264-023-01345-w, PMID: 37651086

